# IMRAS—Immunization with radiation-attenuated *Plasmodium falciparum* sporozoites by mosquito bite: Cellular immunity to sporozoites, CSP, AMA1, TRAP and CelTOS

**DOI:** 10.1371/journal.pone.0256396

**Published:** 2021-08-20

**Authors:** Martha Sedegah, Michael R. Hollingdale, Harini Ganeshan, Maria Belmonte, Jun Huang, Arnel Belmonte, Sandra Inoue, Rachel Velasco, Bradley Hickey, Nimfa Teneza-Mora, Joanne Lumsden, Sharina Reyes, Jo Glenna Banania, Anatalio Reyes, Ivelese Guzman, Thomas L. Richie, Judith E. Epstein, Eileen Villasante

**Affiliations:** 1 Malaria Department, Naval Medical Research Center, Silver Spring, Maryland, United States of America; 2 Henry M. Jackson Foundation, Bethesda, Maryland, United States of America; George Washington University School of Medicine and Health Sciences, UNITED STATES

## Abstract

**Background:**

Immunization with radiation-attenuated sporozoites (RAS) by mosquito bites provides >90% sterile protection against *Plasmodium falciparum* malaria in humans. We conducted a clinical trial based on data from previous RAS clinical trials that suggested that 800–1200 infected bites should induce ~50% protective vaccine efficacy (VE) against controlled human malaria infection (CHMI) administered three weeks after the final immunization. Two cohorts were immunized separately. VE was 55% in Cohort 1 but 90% in Cohort 2, the cohort that received a higher first dose and a reduced (fractional) fifth dose. Immune responses were better boosted by the fractional fifth dose in Cohort 2 and suggested the importance of the fractional fifth dose for increased protection in Cohort 2 responses. Three protected subjects were later boosted and were protected suggesting that protection could be extended to at least 67 weeks.

**Methods:**

The *ex vivo* FluoroSpot assay was used to measure peripheral IFN-γ, IL2, and IFN-γ+IL2 responses to PfNF54 sporozoites and malaria antigens CSP, AMA1, TRAP, and CelTOS using pools of synthetic overlapping 15mer peptides spanning each antigen.

**Results:**

There was no correlation between IFN-γ, IL2, and IFN-γ+IL2 responses to sporozoites and protection, but fold-increases between post-4^th^ and post-5^th^ responses greater than 1.0 occurred mostly in protected subjects. IFN-γ and IL2 responses to TRAP, CelTOS and CSP occurred only in protected subjects. Peripheral IFN-γ, IL2, and IFN-γ+IL2 responses were short-lived and low by 27 weeks post-CHMI but were restored by boosting.

**Conclusions:**

These studies highlight the importance of vaccine dose and schedule for vaccine efficacy, and suggest that CSP, TRAP, AMA1 and CelTOS may be targets of protective immunity. The correlation between fold-increases in responses and protection should be explored in other vaccine trials.

**Trial registration:**

ClinicalTrials.gov NCT01994525.

## Introduction

Radiation-attenuated sporozoites (RAS) induced up to 100% sterile protection against malaria sporozoite challenge in mice [1, 2], non-human primates [3] and up to 93% humans [4, 5], and provided a powerful rationale for development of a malaria vaccine. Since RAS were administered by bite of malaria-infected mosquitoes, a whole organism vaccine was considered impractical, and attention focused on development of subunit vaccines [6]. However, renewed interest in RAS vaccines led to the demonstration that intravenous inoculation of aseptic, purified, cryopreserved, radiation-attenuated *Plasmodium falciparum* sporozoites (PfSPZ Vaccine, Sanaria^®^) elicited 100% sterile protection in humans [7–9]. It is thought that cellular responses directed against hundreds of antigens in liver stages are important mediators of overall protection [7, 8, 10].

To better understand mechanisms of protection and identify protective malaria antigens, we undertook two human trials using *P*. *falciparum* RAS delivered by mosquito bite [11]. In the first trial, 50% vaccine efficacy (VE) was achieved using >1000 infected bites; this was unexpectedly lower than previously obtained despite similar numbers of infected bites used then and previously [11]. This difference in VE may have been due to differences in host genetics, salivary gland sporozoite counts, or time between the final immunization and controlled human malaria infection (CHMI) [11]. Sera and PBMCs from these immunized subjects have been used successfully to identify and characterize novel *P*. *falciparum* antigens as potential candidate vaccines [12, 13].

A new RAS trial (IMRAS: Immunization via Mosquito bite with Radiation Attenuated Sporozoites) was undertaken to reproduce this level of approximately 50% VE in order to generate serum and PBMC samples from both protected and non-protected subjects for analysis of immune mechanisms and antigens associated with protection [14]. We hypothesized that, based on these earlier trials [11], a range of 800–1200 infected bites and an interval of 22–24 days before CHMI might lead to ~50% VE [14]. We recognize that the numbers of immunized subjects in this, and similar earlier, trials are small, but this is a result of the challenges in rearing *P*. *falciparum*-infected mosquitoes and administrating immunization by bite that was reproducible among all subjects. Therefore, IMRAS was not a trial of a RAS-based vaccine for clinical development, but rather an opportunity to further investigate protective immunity resulting from bite of infected mosquitoes as well as to provide serum and PBMC samples to collaborators. As such, analysis of VE and immune mechanisms is based on small numbers of subjects, and we are careful in the interpretation of our results. However, it is unlikely that a clinical trial using immunization by mosquito bite will be performed in the future, and we felt that the information gained from these small Cohorts will be relevant to malaria vaccines in general.

**Fig 1:**
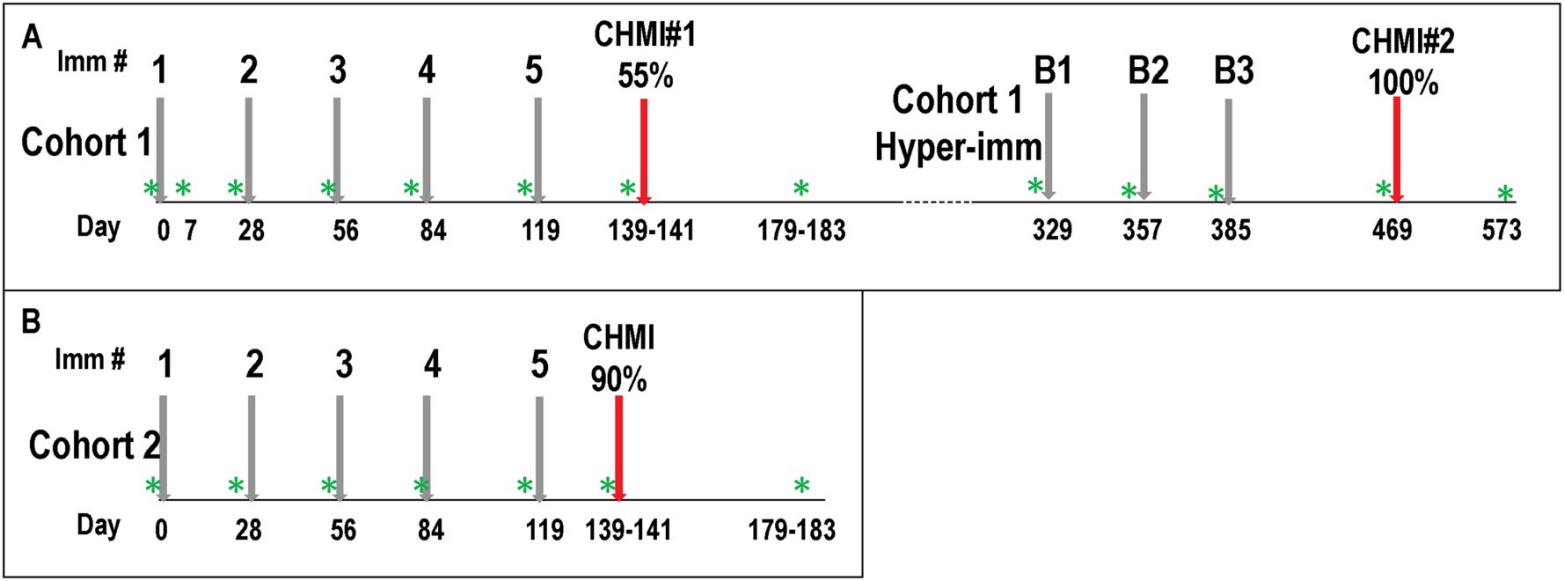
Cohort 1 and Cohort 2 and Cohort 1 hyper-immunized: Flow diagram of immunizations, CHMI and PBMC samples. **Panel A**: Cohort 1 received four monthly immunizations (1, 2, 3, 4) and a fifth immunization (5) 33 days later; 11 subjects received their first CHMI (CHMI#1) 22–24 days post-5^th^; four protected subjects from Cohort 1 were hyper-immunized and received three monthly boosting immunizations (B1, B2, B3) starting 329 days post-1^st^, and three subjects received CHMI#2 on days 472 post-1^st^. Six/11 (55%) immunized subjects were protected against CHMI#1, and three/three (100%) hyperimmunized subjects were protected against CHMI#2. **Panel B**: Cohort 2 followed the same schedule as Cohort 1, and nine/10 (90%) immunized subjects were protected against CHMI. PBMC were taken at times indicated (Green asterisk) that were just prior to each immunization and CHMI, and 38 to 44 days post-CHMI, except an additional sample was taken at 7 days post-1^st^ only in Cohort 1.

IMRAS was performed in two stages (Fig 1) [14]; firstly, a Cohort of 11 subjects (Cohort 1) received approximately similar numbers of RAS infected mosquito bites as in the previous trial [14], and achieved the desired target of 55% VE. This immunization regimen was repeated in an additional 10 subjects (Cohort 2) who received similar total numbers of RAS infected mosquito bites, but VE was 90%. While subjects were not randomized into each Cohort, we attempted to balance these Cohorts according to age and other factors, but we cannot rule out that other differences, that may be unknown, between subjects in each Cohort may have influenced the outcomes. Four protected subjects from Cohort 1 received three additional immunizations (boosts) approximately 27 weeks after the first CHMI. Three of these subjects underwent a second CHMI 12 weeks after their final boost and were each protected, suggesting that protection could be extended with boosting immunizations to at least 67 weeks after the first series of immunizations.

Analysis of the immunization regimens in Cohort 1 and Cohort 2 suggested that VE was not related to total numbers of infected bites or the number of days between the final (fifth) immunization and CHMI [14]. We suggested that two parameters may have affected the differences between Cohorts. Firstly, the numbers of infected bites given after the first immunization were significantly higher in Cohort 2 (median 227, range 194–249) than Cohort 1 (median 175, range 148–264). Since IFN-γ, IL2 and IFN-γ+IL2 responses at 28 days post-1^st^ immunization were lower in Cohort 2 than Cohort 1 [14], it thus remains unclear whether the differences in the first immunization affected VE. However, the numbers of infected bites in the fifth immunization in Cohort 2 (median 77, range 41–96) were significantly lower (1/3^rd^) than the fifth immunization in Cohort 1 (median 239, range 186–272) [14]. This was because the total of infected bites in Cohort 2 after the fourth immunization approached the target number of infected bites; therefore, the numbers of infected bites in the fifth immunization was reduced, resulting in a fractional dose. A reduced final dose also was linked to efficacy of the malaria vaccine RTS,S [15]. Therefore, we particularly investigated whether this reduced (fractional) post-5^th^ dose might affect immune responses and therefore VE.

Here, using peripheral blood mononuclear cells (PBMC) from IMRAS immunized subjects, we measured responses to PfNF54 SPZ and synthetic peptide pools spanning four well characterized candidate malaria vaccine antigens: circumsporozoite protein (CSP), apical membrane antigen-1 (AMA1), thrombospondin related adhesion protein (TRAP), and cell-traversal protein for ookinetes and SPZ (CelTOS). We used the *ex vivo* FluoroSpot assay to detect both interferon-gamma (IFN-γ) and interleukin-2 (IL2) because of its superior sensitivity in detecting low responses [2], as well as our ability to determine which low responses were truly positive [16]. We were interested in differences between protected and non-protected subjects, and differences in Cohort 1 and 2 after each immunization, and especially the influence of the 5^th^ whole (Cohort 1) or fractional dose (Cohort 2).

## Materials and methods

### Ethics

The study was conducted at the Naval Medical Research Center (NMRC) Clinical Trials Center (NMRC CTC) from 2014 to 2016; the CHMIs were conducted at the Walter Reed Army Institute of Research (WRAIR) secure insectary. The study protocol was reviewed and approved by the NMRC Institutional Review Board (IRB) in compliance with all federal regulations governing the protection of human subjects. Both WRAIR and NMRC hold a Federalwide Assurance from the Office of Human Research Protections (OHRP) under the Department of Health and Human Services. NMRC also holds a Department of Defense/Department of the Navy Assurance for human subject protections. All key personnel were certified as having completed mandatory human subjects’ protection curricula and training under the direction of the WRAIR IRB and Human Subjects Protections Branch (HSPB) or the NMRC IRB and Office of Research Administration (ORA). All potential study subjects provided written, informed consent before screening and enrollment and had to pass an assessment of understanding. This study was conducted according to the Declaration of Helsinki as well as principles of Good Clinical Practices under the United States Food and Drug Administration (FDA) Investigational New Drug (IND) application BB-15767. This trial was performed under an IND allowance by the FDA and was registered on ClinicalTrials.gov (NCT01994525).

#### Study subjects

As previously reported [14], enrollment took place at the NMRC CTC from April 2014 until September 2015. Healthy, malaria-naïve, non-pregnant adults between the ages of 18 and 50 were included in this study. Subjects were excluded if they had a history of malaria infection, travel to a malaria endemic region within 6 months of the first immunization, history of long-term residence (> 5 years) in an area known to have significant transmission of *P*. *falciparum*, positive sickle cell screening test, or reactivity by CSP or AMA1 ELISpot assay or ELISA. Subjects were excluded if they had any significant medical condition (cardiovascular, hepatic, renal, pulmonary, or hematological), history of anaphylactic or other severe response to mosquito bites, splenectomy, or evidence of increased cardiovascular risk (defined as >5–10%, 5-year risk) [17]. All females had urine pregnancy test at screening, immediately before each immunization and before CHMI; they were to be excluded from further immunization or CHMI if this was positive. All female subjects agreed to use effective means of birth control for the duration of the trial. The demographics of both cohorts were approximately balanced in gender, age, and ethnic background (S1 Table in S1 Appendix).

### Immune samples

Fresh PBMCs were obtained from subjects in Cohort 1 and 2 pre-immunization; seven days after the first immunization (Cohort 1 only), 28 days after the first, second and third immunizations; 35 days after the fourth immunization; 22–24 days after the fifth immunization (pre-CHMI); and 39–42 days after CHMI (Fig 1). Fresh PBMCs were obtained from Cohort 1 hyperimmunized subjects before each boost and post-CHMI#2.

### *Ex vivo* FluoroSpot IFN-γ, IL2, and IFN-γ+IL2 assay

PBMCs secreting single or multiple cytokines (IFN-γ, IL2 and IFN-γ+IL2) were analyzed by FluoroSpot assay by modifying previously described ELISpot methods [14, 16]. Briefly, 3–4 x 10^5^ PBMCs were suspended in 100 μL complete medium and incubated with an optimized dose of 2.5 x 10^4^ PfSPZ [8]. Additional wells were stimulated with a pool of antigen-specific synthetic peptides (Mimotopes, Clayton, Victoria, Australia) spanning full-length sequences of CSP (GenBank no. X15363), AMA1 (GenBank no. 810891), TRAP (GenBank no. AF249739), and CelTOS (GenBank no. AB194052). A series of 15 amino acid (aa) peptides overlapping by 11 aa were combined into a single pool for each antigen. PBMCs were incubated with each peptide pool at the previously optimized concentration of 1.25 μg/mL. CEF-Class I Peptide Pool Plus (CTL, Ohio, USA) was used as an internal positive control. PHA (leucoagglutinin PHA-1, Millipore Sigma, St. Louis, MO) was used as a positive control for cell viability. Negative control unstimulated PBMC wells received medium only.

Cultures were incubated for 40–42 h at 37°C in 5% CO2 in triplicate and the number of single-staining IFN-γ and IL2-secreting cells and double-staining IFN-γ+IL2-secreting cells were recognized as spot-forming cells (sfc) using an automated FluoroSpot reader (AID iSpot, GmbH, Germany). After removing outliers, the mean number of sfcs of negative control wells was subtracted from the value of each test well. The mean sfcs was expressed as sfcs/10^6^ PBMCs. A positive response was defined as (1) a statistically significant difference (p = ≤0.05) between the average of the number of sfcs in triplicate test wells and the average of triplicate negative control wells (Student’s two tailed t-test), plus (2) at least a doubling of sfcs relative to negative control wells, plus (3) a difference of at least ten sfcs between test and negative control wells.

### Statistical analysis

The non-parametric Mann-Whitney U test was used to compare differences in FluoroSpot responses (between cohorts and between protected and non-protected subjects in each cohort). Statistical significance was defined as a two-tailed P value of ≤0.05. Throughout, changes in responses that meet this definition are reported as significant, and non-significant changes (Mann-Whitney) are descriptively reported for further information.

## Results

### *Ex vivo* FluoroSpot IFN-γ, IL2, and IFN-γ+IL2 responses to PfNF54 SPZ

We previously reported that IFN-γ, IL2, and IFN-γ+IL2 responses to SPZ were significantly higher in Cohort 1 than Cohort 2 after the first and second immunizations, despite the lower vaccine efficacy in Cohort 1 [14]. Here, we have compared responses of protected and non-protected subjects in each Cohort.

#### Cohort 1

Six/11 (55%) subjects were protected, and five/11 (45%) were not protected. After one or more immunizations, IFN-γ, IL2, and IFN-γ+IL2 responses to SPZ occurred in 10/11, 11/11 and 7/11 subjects respectively (S1 Appendix).

**Fig 2:**
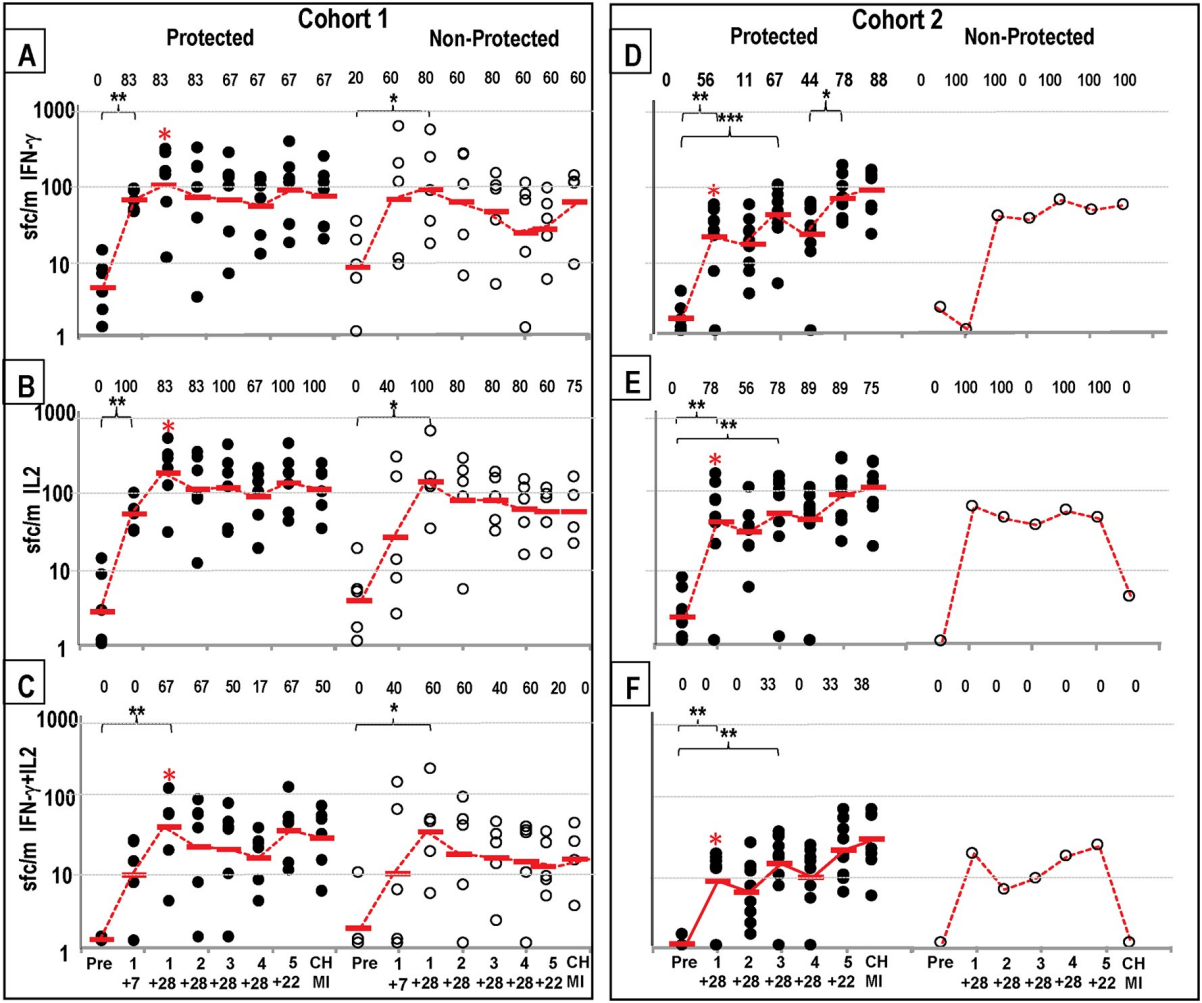
Cohorts 1 and Cohort 2: IFN-γ, IL2 and IFN-γ+IL2 activities to SPZ after each immunization and CHMI. IFN-γ, IL2, and IFN-γ+IL2 responses to SPZ (time points in Fig 1). Protected subjects (closed circles), non-protected subjects (open circles). Geometric means (red bars). Numbers are % positive subjects. Significant changes are shown by *p = <0.05, **p = <0.01, ***p = <0.001. Red asterisk: Time points when differences between Cohort 1 and Cohort 2 were significant (p = <0.05). **Cohort 1: IFN-γ: Panel A: IFN-γ**: responses of protected subjects significantly rose 7 days post-1^st^, peaked 4 weeks post-1^st^, were lowest post-4^th^, rose post-5^th^, and dropped post-CHMI. Responses of non-protected subjects also significantly rose 7 days post-1^st^ and peaked post-1^st^, were unchanged post-5^th^ and rose post-CHMI; % positive subjects followed responses. **Panel B: IL2**: the pattern of IL2 responses and % positive subjects were like IFN-γ responses except for a rise post-CHMI. **Panel C: IFN-γ+IL2**: responses of protected subjects rose more slowly than IFN-γ and IL2 responses, dropped post-4^th^, rose post-5^th^ in protected but not non-protected subjects and dropped post-CHMI. % positive subjects followed this pattern. **Cohort 2: Panel D: IFN-γ**: responses of protected subjects significantly rose 4 weeks post-1^st^, post-3^rd^, significantly rose again post-3^rd^, and significantly rose and peaked post-5^th^ and slightly rose post-CHMI; responses of the single non-protected subject also peaked post-3^rd^ but did not rise post-5^th^. **Panel E: IL2**: responses of protected subjects were like IFN-γ responses, but responses of the non-protected subject declined post-5^th^ and post-CHMI. **Panel F: IFN-γ+IL2**: responses of protected subjects were like IFN-γ responses; responses of the non-protected subject were negative. % positive subjects reflected IFN-γ responses. Red asterisk: Four weeks post-1^st^ IFN-γ, IL2 and IFN-γ+IL2 responses were each significantly higher (p = 0.025; p = 0.03; p = 0.03 respectively) than Cohort 2 post-1^st^ IFN-γ, IL2 and IFN-γ+IL2 responses.

Protected subjects: Geometric mean IFN-γ and IL2 responses (S2 Table in S1 Appendix; Fig 2A and 2B) significantly rose 7 days post-1^st^, peaked 4 weeks post-1^st^, and declined slightly after further immunizations; responses rose post-5^th^ (but the rise was not significant) and dropped post-CHMI. The percent of subjects with positive IFN-γ and IL2 responses was generally highest after the first immunization and in most cases remained high at subsequent time points, varying from 67% to 100%. Double positive (IFN-γ+IL2) responses (S2 Table in S1 Appendix; Fig 2C) were generally 3- to 5-fold lower than responses to IFN-γ or IL2, also significantly rose and peaked 4 weeks post-1^st^ immunization, dropped, and then rose post-5^th^ although this rise was not significant. Percent of subjects with positive double responses varied from 17–67%. Non-protected subjects: geometric mean IFN-γ, IL2, and IFN-γ+IL2 responses (S2 Table in S1 Appendix, Fig 2A–2C) did not significantly rise and peak until 4 weeks post-1^st^, then dropped, and did not rise or rise as steeply post-5^th^. Responses were like protected subjects’ post-1^st^ and post-2^nd^, but were lower post-5^th^ although this drop was not significant. IFN-γ responses rose post-CHMI presumably due to emergence of blood stage parasites. Percent positive subjects followed these responses and similarly peaked 4 weeks post-1^st^.

#### Cohort 2

Nine/10 (90%) subjects were protected, and one/10 (10%) was not protected. After one or more immunizations, IFN-γ and IL2 responses occurred in 9/10 subjects, and IFN-γ+IL2 responses occurred in 5/10 subjects who were all protected (S1 Appendix).

Protected subjects: Geometric mean IFN-γ, IL2, and IFN-γ+IL2 responses (S3 Table in S1 Appendix, Fig 2D–2F) rose significantly post-1^st^, post-3^rd^, and post-5^th^, and rose slightly post-CHMI. The percent of subjects with positive IFN-γ and IL2 responses at the various time points showed a similar pattern. Non-protected subject: IFN-γ responses of the single subject did not become positive until post-2^nd^ whereas IL2 responses became positive post-1^st^ and remained subsequently unchanged and IFN-γ+IL2 responses were negative.

Cohort 1 four-weeks post-1^st^ geometric mean IFN-γ, IL2, and IFN-γ+IL2 responses of protected subjects were significantly higher than protected subjects in Cohort 2 and more subjects were positive (Fig 2).

### Post-5^th^ IFN-γ, IL2, and IFN-γ+IL2 responses to SPZ

#### Post-5^th^ IFN-γ, IL2, and IFN-γ+IL2 responses to SPZ were not predictive correlates of protection

**Fig 3:**
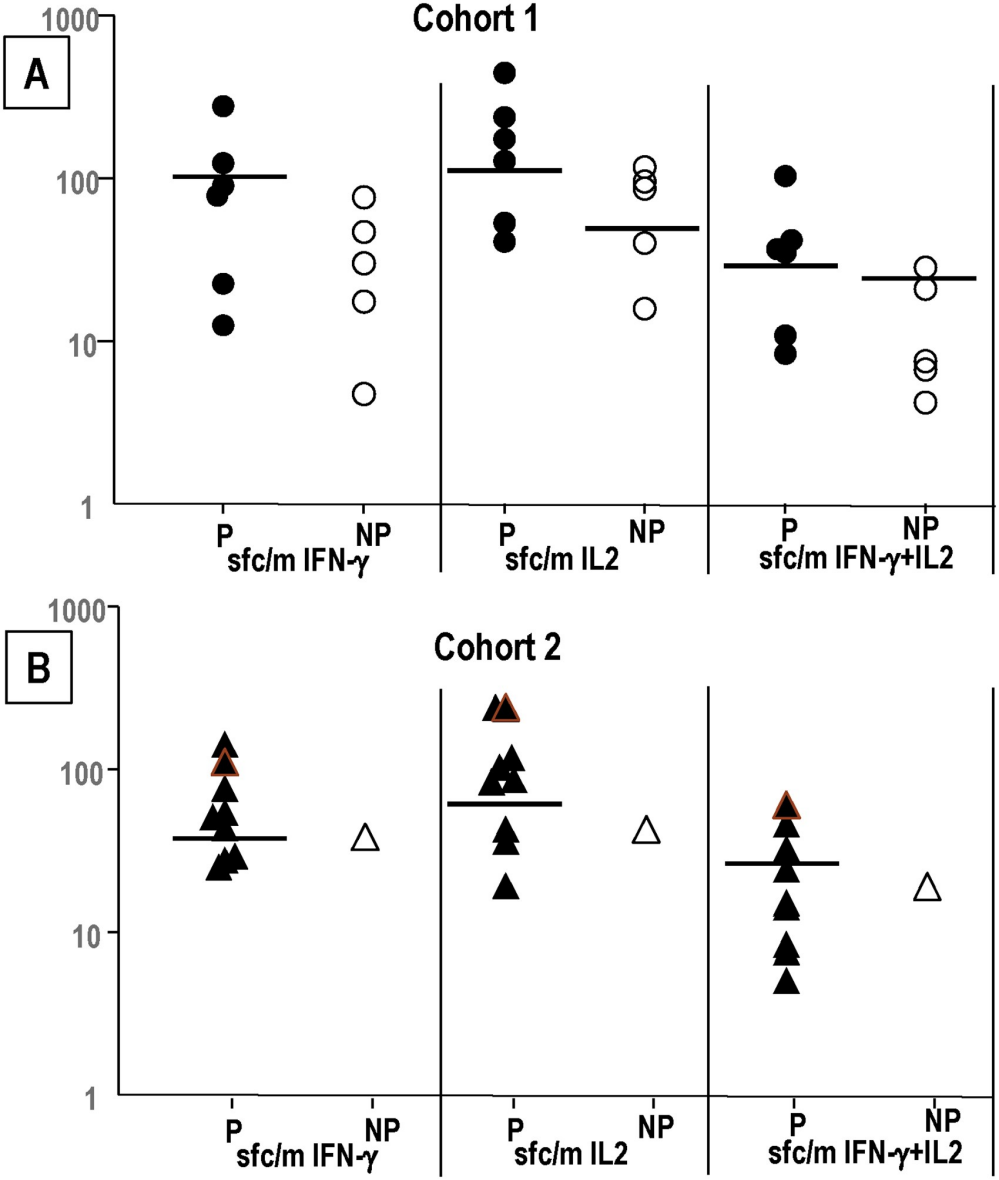
Cohort 1 and Cohort 2: Comparison of post-5^th^ (pre-CHMI) IFN-γ, IL2 and IFN-γ+IL2 responses to SPZ. Cohort 1: protected (n = 6), non-protected (n = 5); Cohort 2: protected (n = 9), non-protected (n = 1). Post-5^th^ (pre-CHMI) Cohort 1 and Cohort 2 IFN-γ, IL2 and IFN-γ+IL2 responses to SPZ of each subject are protected (P) or not protected (NP) to CHMI#1. Horizontal black lines separate subjects with positive (above) or negative (below) responses. **Panel A: Cohort 1:** numbers of protected subjects with higher activities than the highest responding non-protected subject were: IFN-γ (2/6), IL2 (4/6) and IFN-γ+IL2 (4/6). **Panel B: Cohort 2:** numbers of protected subjects with higher activities than the highest responding non-protected subject were: IFN-γ (6/9), IL2 (6/9) and IFN-γ+IL2 (4/9).

Since the fifth dose boosted IFN-γ, IL2, and IFN-γ+IL2 responses that were significant for IFN-γ responses in Cohort 2, we sought to determine whether post-5^th^/pre-CHMI responses were related to protection (Fig 3A and 3B). We separated the responses of protected and non-protected subjects (Fig 3).

Cohort 1 (Fig 3A). 2/6, 4/6 and 4/6 protected subjects had higher post-5^th^ IFN-γ, IL2, and IFN-γ+IL2 responses respectively than all non-protected subjects. Cohort 2 (Fig 3B): 6/9, 6/9 and 4/9 protected subjects had higher post-5^th^ IFN-γ, IL2, and IFN-+IL2 responses respectively than the single non-protected subject. These suggest that the highest IFN-γ, IL2, and IFN-γ+IL2 responses were likely contributing to protection, but do not predict protection as some protected subjects had lower responses than non-protected subjects.

**Fig 4:**
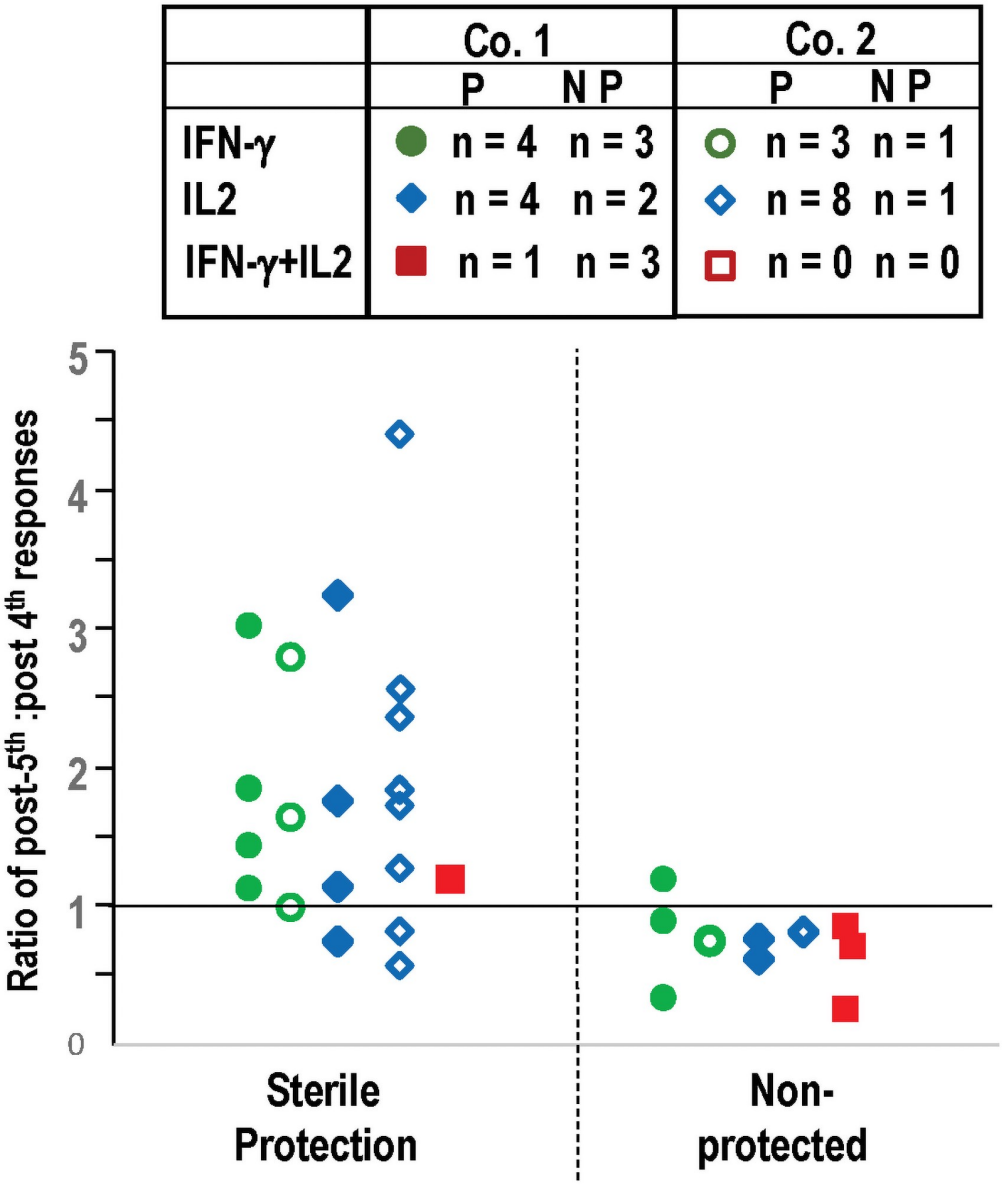
Comparison of the ratios post-5^th^: Post-4^th^ IFN-γ, IL2 and IFN-γ+IL2 responses to SPZ of individual subjects Cohort 1 and Cohort 2. Only subjects with positive post-4^th^ and post-5^th^ IFN-γ, IL2 and IFN-γ+IL2 SPZ responses were included as shown in box. The ratios of post-5^th^: post-4^th^ positive IFN-γ, IL2 and IFN-γ+IL2 responses were determined in protected and non-protected subjects. When responses were unchanged the ratio was 1.0 and that was chosen as an arbitrary cut off. IFN-γ (green) circles, IL2 (blue diamonds) and IFN-γ+IL2 (red squares); Cohort 1 (closed symbols) and Cohort 2 (open symbols). **Protected subjects: IFN-γ**: 7/7 (100%) were 1.0 or greater; **IL2**: 9/12 (75%) were >1.0, **IFN-γ+IL2**: 1/1 (100%) were >1.0. **Non-protected subjects**: **IFN-γ**: 3/4 (75%) were <1.0, **IL2**: 3/3 (100%) were >1.0; **IFN-γ+IL2**: 3/3 (100%) were <1.0. Therefore, only one non-protected subject in Cohort 1 had IFN-γ responses >1.0.

We next determined whether fold-increases post-5^th^ (ratio of post-5^th^: post-4^th^ responses) were related to protection. Unchanged responses resulted in a fold-increase of 1.0, and we distinguished between those with a fold-increase of lower than 1.0, and those with a fold-increase of 1.0 or higher, that was set as an arbitrary cut off. One subject with a value of 1.0 was protected. Only those subjects with positive responses pre- and post-5^th^ were included in this analysis; therefore, the numbers of subjects in this analysis were lower than the total numbers in each cohort and are shown in Fig 4. The actual responses (sfc/m) for each subject are shown in S4 Table in S1 Appendix.

#### The fold-increases of post-5^th^: Post-4^th^ IFN-γ, IL2, and IFN-γ+IL2 responses to SPZ are predictive correlates of protection

Protected subjects: Post-5^th^ fold increases of IFN-γ, IL2, and IFN-γ+IL2 responses of protected subjects in both Cohorts were 1.0 or higher in 7/7 (100%), 9/12 (75%) and 1/1 (100%) subjects meeting positivity criteria, respectively (Fig 4). Non-protected subjects: post-5^th^ fold increases of IFN-γ, IL2, and IFN-γ+IL2 responses of the non-protected subjects were lower than 1.0 in 3/4 (75%), 3/3 (100%) and 3/3 (100%) subjects meeting positivity criteria, respectively (Fig 4). We conclude that rising FluoroSpot IFN-γ responses following the 5^th^ immunization led to protection in every case, although one non-protected subject in Cohort 1 had a ratio of 1.2 and could be considered a false positive. When subjects with responses that met positive criteria at either post-4^th^ or post-5^th^, but not both, are included, the same association is seen (S1 Appendix).

These data suggest that after the 5^th^ dose, the fold increases of IFN-γ, IL2, and/or IFN-γ+IL2 responses are highly associated with protection to CHMI in subjects with positive responses (p<0.002, Mann-Whitney U test).

**Fig 5:**
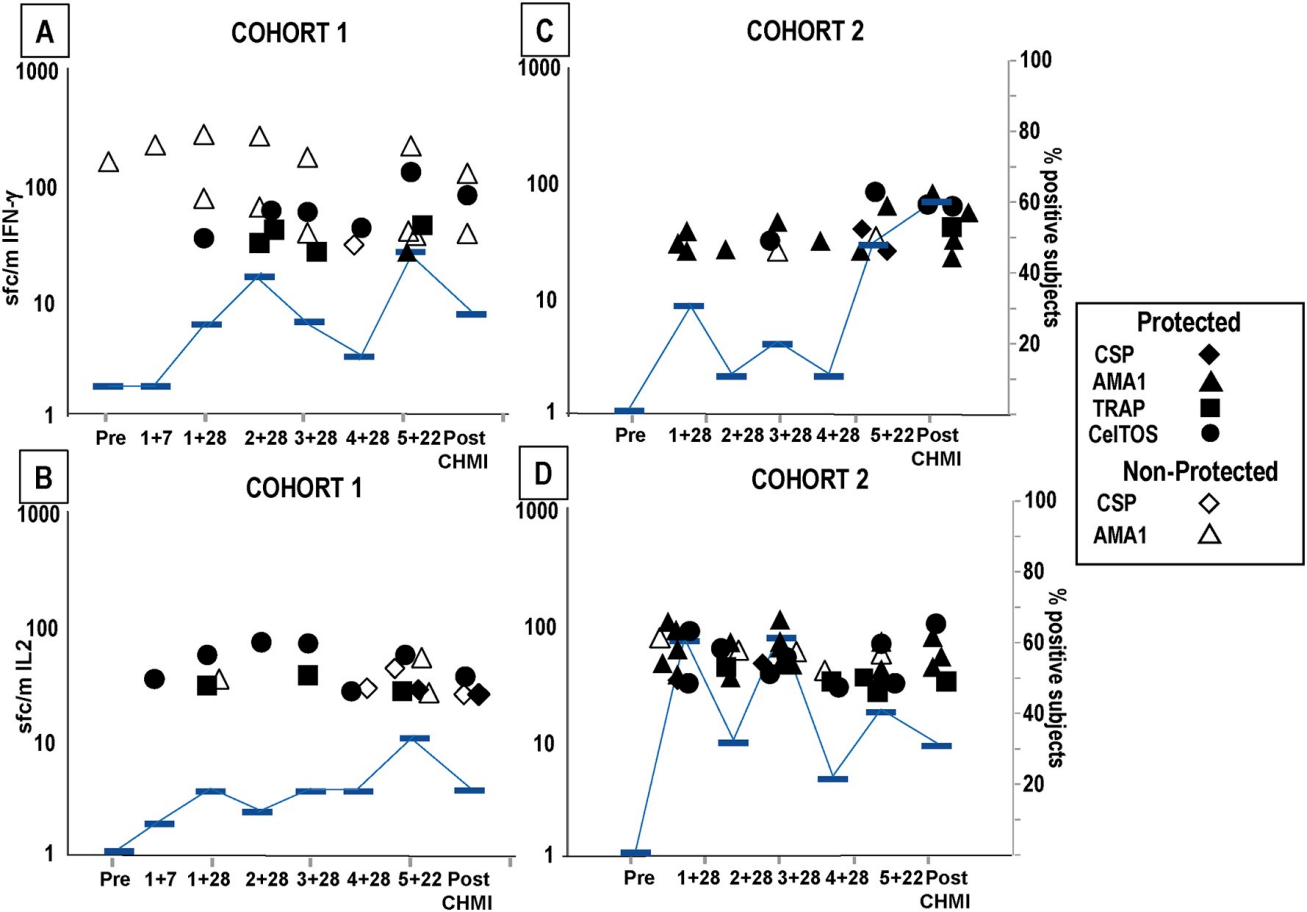
Cohort 1 and Cohort 2: IFN-γ and IL2 activities to CSP, AMA1, TRAP and CelTOS after each immunization and CHMI. IFN-γ and IL2 responses to CSP (diamonds), AMA1 (triangles), TRAP (squares) and CelTOS (circles) at pre-immunization (Pre), one week (1*, Cohort 1 only) and four weeks post-1^st^ (1), post-2^nd^ (2), post-3^rd^ (3), five weeks post-4^th^ (4), 22–24 days post-5^th^ (5) immunizations, and 39–42 days post-CHMI (Post-CHMI). Protected subjects (closed symbols), non-protected subjects (open symbols). Percent of positive subjects, defined as any subject who had positive responses to one or more of the 4 antigens (blue bars). Some subjects were positive to more than one antigen. **Panel A: Cohort 1: IFN-γ**: responses peaked post-2^nd^, dropped by post-4^th^, rose post-5^th,^ and dropped post-CHMI. The only two subjects who had had IFN-γ responses to TRAP and CelTOS were both protected, and one protected subject had low responses to AMA1 post-5^th^; three non-protected subjects had responses to CSP and AMA1. **Panel B: Cohort 1: IL2**: numbers of positive subjects remained low until they rose post-5^th^; two protected subjects had IL2 responses to TRAP and CelTOS, and one protected subject had low responses to CSP post-5^th^; three non-protected subjects had responses to CSP and AMA1. **Panel C: Cohort 2: IFN-γ**: responses rose post-1^st^, dropped, and then rose post-5^th^ and post-CHMI; two protected subjects had responses to CSP, AMA1, CelTOS, and two additional subject had responses to CSP post-5^th^; the non-protected subject only had responses to AMA1. **Panel D: Cohort 2: IL2**: more subjects had positive responses than IFN-γ responses, were variable during immunization and post-CHMI, and peaked post-1^st^ and post-3^rd^; seven protected subjects had IL2 responses to CSP, AMA1, TRAP and CelTOS, whereas the non-protected subject only had responses to AMA1.

We next investigated the antigen-specificity of these responses. IFN-γ, IL2, and IFN-γ+IL2 responses to SPZ are likely recognizing hundreds to thousands of malaria antigens. As a first step to understanding the contributions of antigen-specific responses, we analyzed IFN-γ, IL2, and IFN-γ+IL2 responses to four lead malaria vaccine antigens expressed by the pre-erythrocytic parasite stages: CSP, AMA1, TRAP and CelTOS (Fig 5).

### IFN-γ and IL2 responses to CSP, AMA1, TRAP and CelTOS peptides

Responses of all subjects in Cohort 1 and Cohort 2 to CSP, AMA1, TRAP, and CelTOS peptides during immunization and post-CHMI are shown in S1 and S2 Figs in S1 Appendix. Only IFN-γ and IL2 antigen-specific responses were positive, and IFN-γ+IL2 responses were negative in all subjects.

#### Cohort 1

Six/11 (55%) subjects had positive (IFN-γ and/or IL2) responses against one or more antigens at some point during immunization, and five/11 (45%) subjects were negative throughout (S1 Appendix). IFN-γ and/or IL2 responses were more frequent to AMA1 (4/11, 36%) than CSP (3/11, 27%), TRAP (2/11, 18%) or CelTOS (2/11, 18%); responses to TRAP and CelTOS were only present in 2/6 protected subjects and absent in non-protected subjects. We then compared IFN-γ and IL2 responses after each immunization.

One subject had positive IFN-γ responses to AMA1 pre-immunization and 7 days post-1^st^ and subsequently. No other subjects had positive IFN-γ responses at 7 days post-1^st^; responses to one or more antigens were detected 4 weeks post-1^st^, rose post-2^nd^, dropped post-4^th^, peaked post-5^th,^ and dropped post-CHMI (Fig 5A). Therefore, IFN-γ responses to antigens developed more slowly than to SPZ, but IFN-γ responses to antigens and SPZ both declined post-4^th^. Fewer subjects had IL2 responses than IFN-γ responses; these also peaked post-5^th^ and dropped post-CHMI (Fig 5B).

#### Cohort 2

Eight/10 (80%) subjects had positive IFN-γ and/or IL2 responses against one or more antigens at some point during immunization, and 2/10 (20%) subjects were negative throughout (S1 Appendix). Therefore, during immunization, subjects with responses to these antigens were marginally more frequent than Cohort 1, IFN-γ and/or IL2 responses were most frequent to AMA1 (8/10, 80%) with fewer to CelTOS (3/10, 30%), CSP (3/10, 30%) and TRAP (2/10, 20%), and were therefore like Cohort 1 where responses to AMA1 were also most frequent.

Positive IFN-γ responses to one or more antigens were detected 4 weeks post-1^st^, dropped post-4^th^, peaked post-5^th,^ and rose post-CHMI (Fig 5C) and resembled IFN-γ responses to SPZ. More subjects had IL2 responses than IFN-γ responses; these peaked post-1^st^ and post-3^rd^, rose post-5^th,^ and dropped post-CHMI (Fig 5D) and resembled IL2 responses to SPZ. The non-protected subject only had IFN-γ responses to AMA1. Responses in Cohort 2 to these antigens were more frequent than Cohort 1.

We next explored whether the boosting observed in IFN-γ and IL2 responses to SPZ and individual antigens post 5^th^ immunization were related.

### Subjects with higher post-5^th^ IFN-γ and IL2 responses to SPZ also have higher positive IFN-γ and IL2 responses to TRAP and CelTOS

**Fig 6:**
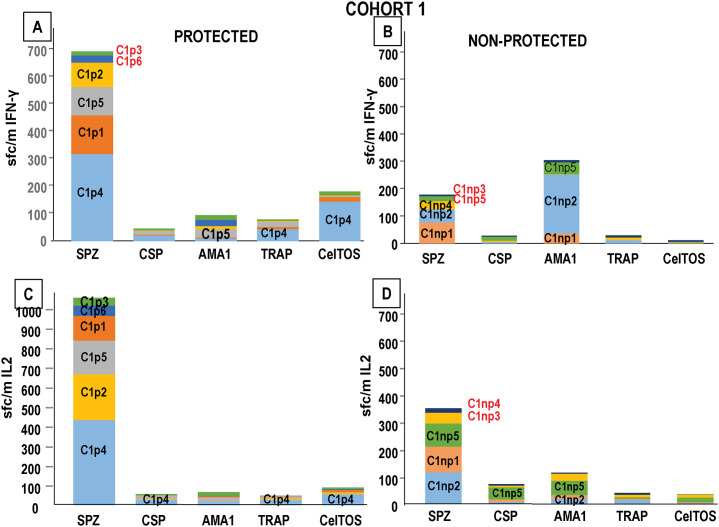
Cohort 1: Higher post-5^th^ responses to SPZ are linked to post-5^th^ IFN-γ and IL2 responses to CSP, AMA1, TRAP and CelTOS. Cohort 1: post-5^th^ IFN-γ and IL2 responses to SPZ, CSP, AMA1, TRAP and CelTOS (C1p = Cohort 1 protected; C1np = Cohort 1 not protected) are color-coded. Subjects with negative SPZ responses are shown in red font. **Panel A: Protected**: **IFN-γ**: the highest SPZ IFN-γ responder (C1p4) had IFN-γ responses to TRAP and CelTOS; the third highest SPZ IFN-γ responder (C1p5) had IFN-γ responses to AMA1. The remaining protected subjects were negative to each antigen. **Panel B: Non-protected**: **IFN-γ**: the three highest SPZ IFN-γ responders (C1np1, C1np2) had IL2 responses only to AMA1. **Panel C: Protected**: **IL2**: the highest SPZ IL2 responder (C1p4) had IL2 responses to CSP, TRAP and CelTOS. The remaining protected subjects were negative with each antigen. **Panel D: Non-Protected**: **IL2**: the third and fourth highest SPZ IL2 responders (C1np5, C1np2) had IL2 responses to CSP (C1np5) and AMA1 (C1np5, C1np2).

**Fig 7:**
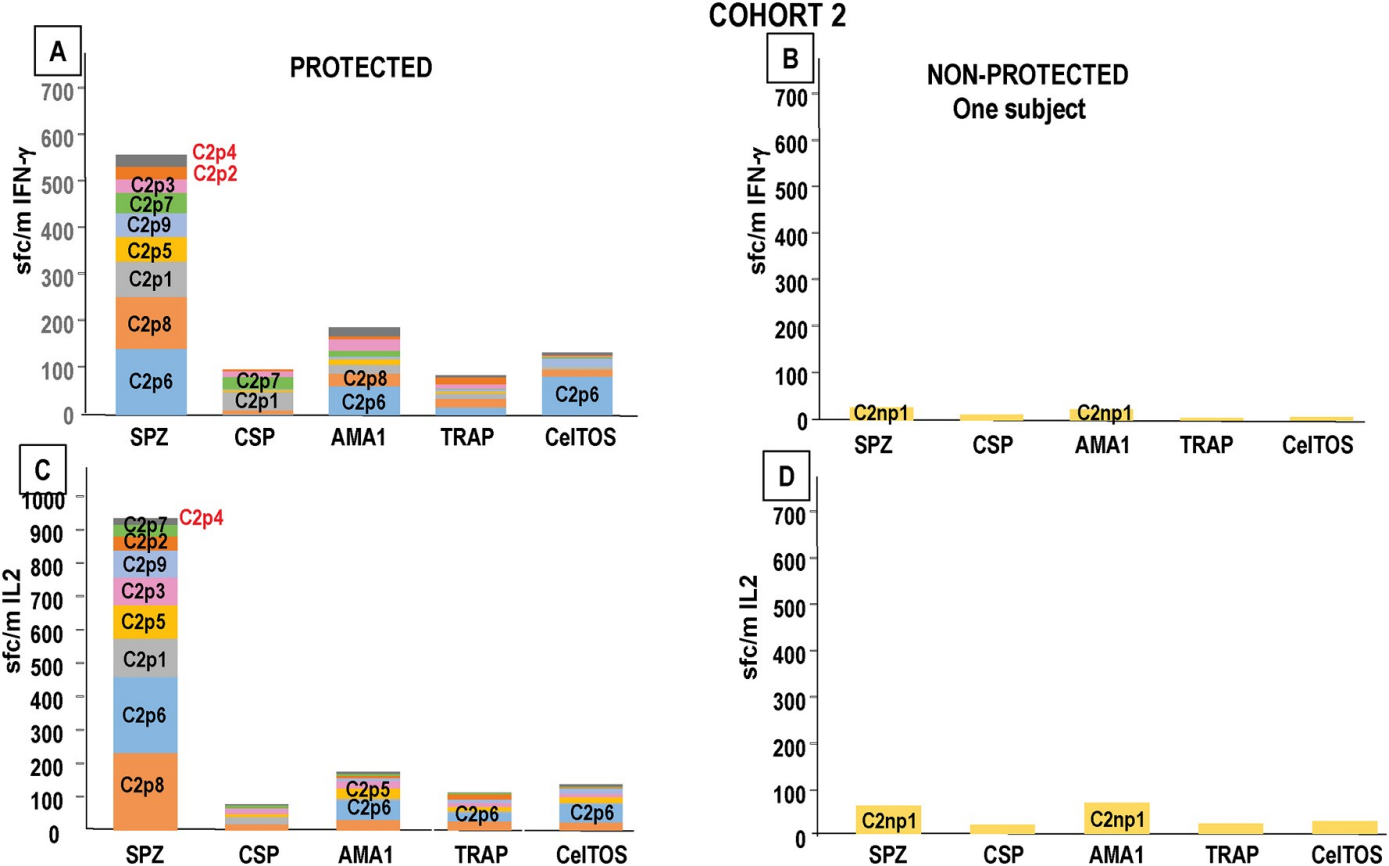
Cohort 2: Higher post-5^th^ protective responses to sporozoites are linked to post-5^th^ protective IFN-γ and IL2 responses to CSP, AMA1, TRAP and CelTOS. Cohort 2: post-5^th^ IFN-γ and IL2 responses to SPZ, CSP, AMA1, TRAP and CelTOS (C2p = Cohort 2 protected; C2np = Cohort 2 not protected) are color-coded; subjects with negative responses are shown in red. **Panel A: Protected**: **IFN-γ**: the highest SPZ IFN-γ responder (C2p6) had IFN-γ responses to AMA1 and CelTOS; the second highest SPZ IFN-γ responder (C2p8) had IFN-γ responses to AMA1; the third and sixth highest SPZ IFN-γ responders (C2p1, C2p7) had IFN-γ responses to CSP. The remaining five protected subjects were negative with each antigen. **Panel B: Non-protected**: **IFN-γ**: the non-protected protected subject (C2np1) had low positive responses to sporozoites and IFN-γ responses to AMA1. **Panel C: Protected**: **IL2**: the two highest SPZ IL2 responders (C2p8, C2p6) had IL2 responses to AMA1, TRAP and CelTOS and the fourth highest SPZ IL2 responder (C2p5) had IL2 responses to AMA1. The remaining six protected subjects were negative with each antigen. **Panel D: Non-protected**: **IL2**: the non-protected subject (C2np1) had IL2 responses to sporozoites and AMA1.

Post-5^th^ IFN-γ and IL2 responses to SPZ of protected and non-protected subjects that were ranked high to low in Fig 3 were then compared to post-5^th^ IFN-γ responses to individual antigens (Figs 6 and 7).

#### Cohort 1

The protected subjects with the highest and third highest IFN-γ responses to sporozoites also had positive IFN-γ responses to TRAP and CelTOS and AMA1, respectively (Fig 6A). The five non-protected subjects were lower or negative sporozoite responders (Fig 6B) and three had IFN-γ responses only to AMA1. The protected subject with the highest IL2 response to sporozoites (same as the highest IFN-γ responder) was positive to CSP, TRAP and CelTOS (Fig 6C), whereas the lower non-protected sporozoite responders were positive with CSP and AMA1 (Fig 6D).

#### Cohort 2

Four of the highest IFN-γ sporozoite responders had positive IFN-γ responses to CSP, AMA1, and CelTOS (Fig 7A), whereas the single non-protected subject had a low IFN-γ response to SPZ and AMA1 (Fig 7B). Eight/9 protected subjects had IL2 responses to AMA1, TRAP and CelTOS (Fig 7C), and the single non-protected subject had an IL2 response to AMA1 (Fig 7D).

Therefore, two of the higher IFN-γ and IL2 protected sporozoite responders in Cohort 1, and five higher IFN-γ and IL2 protected sporozoite responders in Cohort 2, had IFN-γ and IL2 responses to CSP, AMA1, TRAP or CelTOS, whereas the lower non-protected responders had IFN-γ and IL2 responses only to AMA1. Since responses to individual antigens were low compared to responses to SPZ, and three/six protected subjects in Cohort 1 and two/nine protected subjects in Cohort 2 did not have IFN-γ or IL2 to any of these four tested antigens, it is likely that responses to other antigens were associated with protection.

### Cohort 1 hyper-immunization: Protection and IFN-γ, IL2, and IFN-γ+IL2 responses to SPZ and individual antigens

In Cohort 1, four protected subjects (three high and one low sporozoite responders) received three monthly boosts (B1, B2 and B3). Three of these subjects received a second CHMI 12 weeks after the final boost and all three were fully protected.

#### Sporozoites

**Fig 8:**
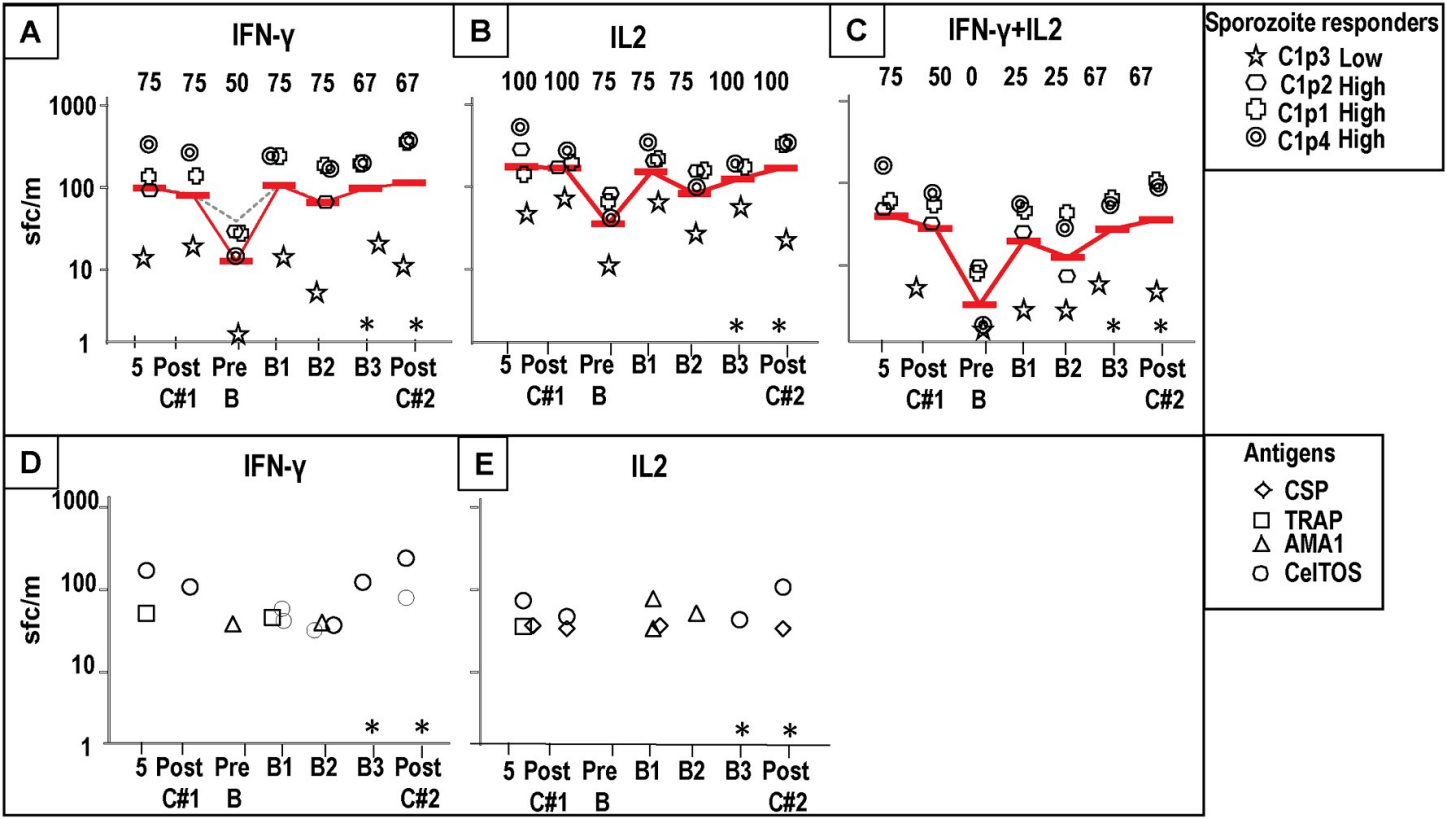
Cohort 1 hyperimmunized subjects: IFN-γ, IL2 and IFN-γ+IL2 responses to sporozoites, CSP, AMA1, TRAP and CelTOS. IFN-γ, IL2 and IFN-γ+IL2 responses of four subjects protected to CHMI#1 were measured after post-5^th^ (5) immunization, 39–41 days post-CHMI#1 (Post-C#1), 27 weeks post-CHMI#1/pre-boost (PreB), post-1^st^ boost (B1), post-2^nd^ boost (B2), post-3^rd^ boost (B3) and 15 weeks post-CHMI#2 (Post-C#2). Responses to sporozoites: three high responders (C1p1 stars; C1p2 rounded squares; C1p4 crosses) and one low responder (C1p3 double circles); geometric mean of sporozoite responses (red bar); numbers at top of Panel are % positive subjects. *Only 3 subjects (C1p2; C1p3, C1p4) were tested at these time points. **Panel A: IFN-γ responses: sporozoites**: IFN-γ responses declined by 27 weeks after CHMI#1/pre-boost but rose post-1^st^ boost and were then largely unchanged. **Panel B: IL2 responses: sporozoites**: IL2 responses followed the same pattern as IFN-γ responses. **Panel C: IFN-γ+IL2 responses: sporozoites**: IFN-γ+IL2 responses disappeared by 27 weeks after CHMI#1/pre-boost, and slowly rose post-1^st^, and post-3^rd^ boosts and post-CHMI#2. Responses to antigens: CSP (diamonds), AMA1 (triangles), TRAP (squares) and CelTOS (circles). **Panel D: IFN-γ**: only one subject (C1p3) had a positive IFN-γ response to AMA1 by 27 weeks post-CHMI#1/pre-1^st^ boost; post-1^st^ boost and post-2^nd^ boost IFN-γ responses to individual antigens rose. **Panel E: IL2**: no subjects had IL2 responses to any antigen by 27 weeks after CHMI#1/pre-1^st^ boost; post-1^st^ boost and post-2^nd^ boost IFN-γ responses to individual antigens rose.

By pre-B1, 27 weeks post-CHMI #1, IFN-γ and IL2 responses to SPZ had declined (Fig 8A and 8B) and IFN-γ+IL2 responses to SPZ had disappeared (Fig 8C). B1 raised IFN-γ and IL2 responses to levels that were like post-5^th^/pre-CHMI levels and were largely maintained after the following boosts and CHMI. IFN-γ+IL2 responses did not peak until post-B3. The subjects that had higher or lower responses pre-B1 remained high or low responders after each boost. Therefore, the boost immunizations restored IFN-γ, IL2 and IFN-γ+IL2 responses to those associated with protection to CHMI #1.

#### CSP, AMA1, TRAP and CelTOS

By pre-B1, 27 weeks post-CHMI#1, only one subject had an IFN-γ response to AMA1, and IL2 responses had disappeared. However, during the three boosts responses were restored to post-5^th^ activities (Fig 8D and 8E) that were associated with protection.

## Discussion

The design of this phase 1 clinical trial was based on a prediction from previous RAS trials that 800–1200 infected bites would achieve 50% efficacy against a CHMI performed three weeks after the final immunization [14]. In Cohort 1 we indeed achieved 55% VE, but when repeated in Cohort 2, we achieved 90% VE, despite the similar total numbers of infected bites [14]. We found it was difficult to control both the sporozoite load in mosquito salivary glands and the number of infectious mosquito bites achieved during a biting session, and unintentionally achieved a higher rate of infectious bites in the second cohort during the first and subsequent immunizations, requiring a reduced final dose in order to not exceed the target total number of infectious bites. The major vaccination parameters differentiating Cohorts 1 and 2 were the significantly lower number of infected bites in the first immunization in Cohort 1 compared to Cohort 2, the significantly reduced (three-fold) fifth dose in Cohort 2 compared to Cohort 1, and the significantly lower sporozoite gland scores in Cohort 1 than Cohort 2 during immunization except during the first immunization [14].

IFN-γ, IL2 and IFN-γ+IL2 responses to SPZ by FluoroSpot assay were significantly higher in Cohort 1 compared to Cohort 2 post-1^st^ immunizations, despite the lower numbers of infected bites and lower gland scores in Cohort 1 [14], although pre-CHMI, IFN-γ responses were similar between the two groups. We found, however, that IFN-γ responses significantly rose, and IL2 and IFN-γ+IL2 responses rose (but not significantly) post-5^th^ in Cohort 2, and rose, but not significantly, in Cohort 1, suggesting that the reduced fifth dose in Cohort 2 may have increased cellular responses and therefore efficacy. Here, we examined these responses in more detail, particularly those of protected and non-protected subjects and we also measured responses to four individual lead malaria candidate antigens.

In Cohort 1, the number of infected bites in the first immunization was significantly lower than Cohort 2, as mentioned above. The numbers of mosquito gland SPZ were similar in both Cohorts, and if we assume that the numbers of SPZ injected by each bite are similar, it is possible that more SPZ were injected in Cohort 2 than Cohort 1. However, post-1^st^ FluoroSpot IFN-γ, IL2, and IFN-γ+IL2 responses were significantly higher in Cohort 1 than Cohort 2, suggesting an inverse relationship of cellular responses and dose. Others have suggested that antigen dose influences TCR-stimulation and regulates Th1/2 polarization, and antigen concentration affects CD8+ T cell avidity [18]. Further studies are required to determine the effects of antigen dose (numbers of SPZ delivered by bite) on cellular responses. However, it has been well established that there is a relationship between antigen dose and humoral responses [19] consistent with the higher antibody activities measured by ELISA to CSP in Cohort 2 than Cohort 1 [14]. However, it is possible that the timing of samples may have also affected this comparison, as cellular responses may have developed before antibody responses after each immunization, and 28-day samples may have favored antibody responses. Cellular responses after the first immunization were detected at 7 days but rose further by 28 days. Therefore, the immune response timing remains to be further elucidated.

Another important parameter may be post-5^th^ immunization IFN-γ, IL2, and IFN-γ+IL2 responses. IFN-γ responses increased slightly and non-significantly in Cohort 1 but more vigorously and significantly in Cohort 2, suggesting that the reduced (fractional) 5^th^ dose in Cohort 2 better boosted responses. Moreover, the boosting effect of the 5^th^ dose in both cohorts, expressed as the fold-increase compared to post-4^th^ responses rather than levels of post-5^th^ responses, was significantly associated with protection.

While we do not envisage that the five-dose IMRAS regimen will undergo further clinical evaluation, we suggest that the concept of fractional dosing, as well as a delay in the final immunization, may improve vaccine efficacy is becoming better established in malaria and other diseases [20–24]. Recently, a clinical trial of the malaria vaccine RTS,S, usually given in three doses monthly, showed that a delayed fractional dose regimen where the final dose was 20% of the full dose and administered after a six-month interval gave a significant increase in vaccine efficacy [15]. The original RTS,S vaccine trial also used this delayed fractional regimen with similarly high vaccine efficacy [25]. The mechanisms by which a fractional dose and/or delay in immunization influences immune maturation [26] and vaccine efficacy requires further investigation, particularly to understand the role of cellular responses and whether fractional dosing increased the numbers of liver-resident memory T cells. This may be directly applicable to the malaria vaccine candidate PfSPZ Vaccine that contains irradiated, aseptic, purified, cryopreserved PfSPZ administered intravascularly by rapid direct venous inoculation (DVI) [8, 27].

Immunization with RAS induces sterile immunity to sporozoite challenge that is by T cells against antigens expressed in liver-stage malaria in the context of MHC molecules on the surface of an infected hepatocyte [7, 8, 10, 28, 29]. Liver-resident memory T cells (T_RM_) are crucial, do not recirculate and are associated with production of IFN-γ or other cytokines [30]. T_RM_ cells patrol liver sinusoids and cluster around infected hepatocytes [31, 32] and have a half-life of about 28 days [30], and multiple immunizations can induce larger numbers of T_RM_ cells [33], and it is likely that T_RM_ cell precursors can directly access liver T_RM_ cell niches during blood circulation. If this occurs in humans, we suggest that analysis of peripheral responses may be relevant to understanding liver T_RM_ responses that are normally not accessible. However, liver resident memory T cells have been studied in humanized mice [34] and identified using transcriptional analyses [35].

The immunodominant CSP has been widely studied, but RAS immunization can induce sterile protection in the absence of responses to CSP, establishing that multiple SPZ or liver stage antigens are likely involved [36, 37]. Earlier studies using RAS-immunized subjects from clinical trials identified other antigens such as CelTOS, originally called Antigen 2 [13]. We used a panel of four lead malaria vaccine candidate antigens (CSP, AMA1, TRAP and CelTOS) to examine these responses and found that IFN-γ and IL2 responses to TRAP and CelTOS in Cohort 1 and to CSP, TRAP and CelTOS in Cohort 2 occurred in many of the protected volunteers, but not in non-protected subjects. The sample size is too small to confidently say that RAS immunization induced VE associated with CSP, TRAP and CelTOS but not AMA1, especially as potentially hundreds of other antigens may be involved. But these outcomes do lend further support to using these antigens in subunit malaria vaccines. In addition, some higher ranked IFN-γ responders from both cohorts that were protected did not have responses to these antigens, consistent with the role of many sporozoite antigens in protection. We are exploring the HLA-restriction of these responses to better understand their association with protection and particularly the importance of genetically restricted epitopes.

A major goal of malaria vaccines is to establish a regimen with a maximum of 3 doses that induces protection in at least 80% of subjects that is durable and protects against multiple strains. Such a vaccine would meet the goals of the World Health Organization (WHO) Malaria Vaccine Technology Roadmap. A subset of protected subjects from Cohort 1 were hyperimmunized and were all protected against a second CHMI at 47 weeks after their previous CHMI. The magnitude of peripheral IFN-γ, IL2, and IFN-γ+IL2 responses had significantly declined by 29 weeks after the first CHMI, but IFN-γ and IL2 responses were restored after one boost and IFN-γ+IL2 after three boosts. Significant protection is durable up to 28 months in the absence of a boost following immunization with chemo-attenuated SPZ [38]. More studies are needed to establish the need for and optimal timing of boosting in whole SPZ immunization regimens, and whether boosting will achieve and maintain the target of 80% protection.

A major limitation of this study is the small group sizes and variation within each group, so we have been cautious in our interpretation of the outcomes and have used them to particularly hypothesize about fractional dosing especially for malaria vaccines A second constraint to these studies and other such similar clinical trials is that only peripheral cellular responses can be measured, whereas it is likely that liver-resident memory CD8+ T cells (T_RM_) are essential for protection, as shown in mice [33, 35, 39] and non-human primates [8]. Experimental data and mathematical modeling have suggested that the spleen is critical during priming and the local reactivation of T_RM_ that occurs during boosting [40]. Therefore, prime-and-trap vaccination strategies that exploit these mechanisms may be useful and could be explored with RAS or other whole organism vaccines.

## Supporting information

S1 Appendix (DOCX)
