# Supplementary material for: IMRAS—Immunization with radiation-attenuated Plasmodium falciparum sporozoites by mosquito bite: Cellular immunity to sporozoites, CSP, AMA1, TRAP and CelTOS
Source: PLoS One. 2021 Aug 20;16(8):e0256396. doi: 10.1371/journal.pone.0256396 (PMC8378721; doi:10.1371/journal.pone.0256396)
Supplement: S1 Appendix — (DOCX) [file pone.0256396.s001.docx]

**S1 Appendix**

**IMRAS: Immunization with Radiation-Attenuated *Plasmodium falciparum* Sporozoites by mosquito bite: cellular immunity to sporozoites, CSP, AMA1, TRAP and CelTOS**

**S1 Fig. Cohort 1: IFN-γ, IL2, and IFN-γ + IL2 responses to sporozoites, CSP, AMA1, TRAP, and CelTOS**

Subjects are grouped as protected or not protected after CHMI. Subjects were tested at seven days after 1^st^ (1*) and four weeks after each (1, 2, 3, 4, 5) immunization and post-CHMI#1 (C). Positive responses are color-coded: IFN-γ (blue), IL2 (red), IFN-γ+IL2 (green). Subjects not tested (gray). C1p=Cohort 1 protected; C1np=Cohort 1 not protected.

**Protected to CHMI.** C1p1 – C1p6: all 6 subjects developed IFN-γ and IL2 responses, and 4/6 developed IFN-γ+IL2 responses to sporozoites. Protected subjects had IFN-γ responses only to AMA1 (C1p5), TRAP (C1p1), and TRAP and CelTOS (C1p4). One protected subject had IL2 responses to CSP, TRAP, and CelTOS (C1p4); one protected subject had IL2 responses to CelTOS (C1p1) and one protected subject had IL2 responses to CSP post-CHMI (C1p3). IFN-γ and IL2 responses to TRAP and CelTOS were absent in non-protected subjects. There were no IFN-γ+IL2 responses to any antigen.

**Not protected to CHMI.** C1np1 – C1np5: 4/5 subjects developed IFN-γ responses, all subjects developed IL2 responses and 3/5 subjects developed IFN-γ+IL2 responses to sporozoites. 3/5 subjects (C1np1, C1np2, C1np5) had IFN-γ responses to AMA1 that also occurred in one protected subject, and one subject (C1np2) had IFN-γ responses to CSP that were absent in protected subjects. In addition, 3/5 subjects had IL2 responses to AMA1 only (C1np1), and CSP and AMA1 (C1np2, C1np5). There were no IFN-γ+IL2 responses to any antigen.

**S2 Fig. Cohort 2: IFN-γ, IL2 and IFN-γ responses to sporozoites, CSP, AMA1, TRAP and CelTOS**


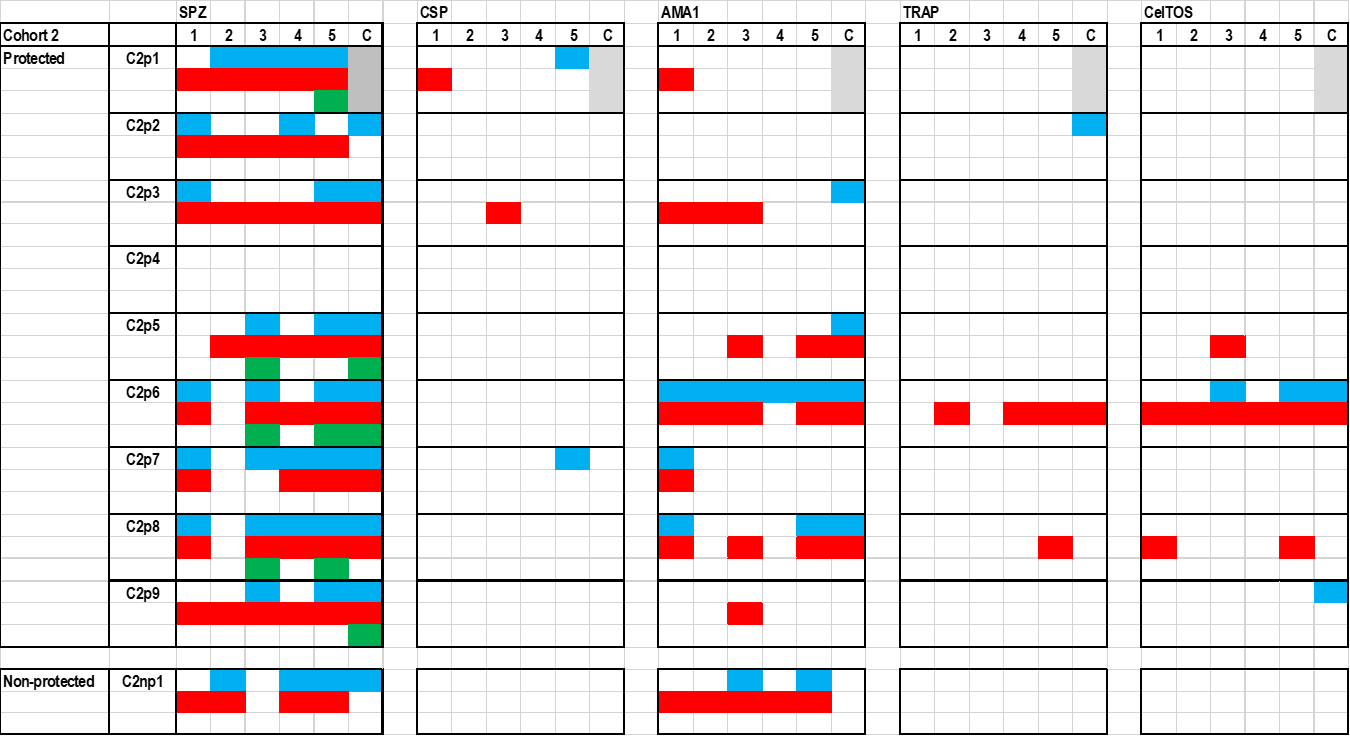


Subjects are grouped according to outcomes after CHMI as protected or not protected. Subjects were tested at four weeks after each dose of PfSPZ (1, 2, 3, 4, 5), and post-CHMI#1 (C). Positive responses are color-coded: IFN-γ (blue), IL2 (red), IFN-γ+IL2 (green). Subjects not tested (gray).

**Protected to CHMI.** C2p1– C2p9: 8/9 subjects had IFN-γ and IL2 responses, and 5/9 had IFN-γ+IL2 responses to sporozoites. One protected subject (C2p4) did not develop measurable responses to sporozoites or antigens. Protected subjects had IFN-γ responses only to CSP (C2p1) and AMA1 (C2p8), both CSP and AMA1 (C2p7), and both CelTOS and AMA1 (C2p6). In addition, protected subjects developed IL2 responses only to AMA1 (C2p7, C2p9), to CSP and AMA1 (C2p1, C2p3), to AMA1 and CelTOS (C2p5), and to AMA1, TRAP and CelTOS (C2p6, C2p8). There were no IFN-γ+IL2 responses to any antigen.

**Not protected to CHMI.** C2np1: this subject developed IFN-γ and IL2 responses but not IFN-γ+IL2 responses to sporozoites, and IFN-γ and IL2 responses to AMA1 that also occurred in 7 protected subjects. The non-protected subject did not develop IFN-γ+IL2 responses to any antigen.

**S3 Fig: Comparison of the ratios post-5^th^: post-4^th^ IFN-γ, IL2 and IFN-γ+IL2 responses to SPZ of individual subjects Cohort 1 and Cohort 2**

Subjects with positive post-4^th^ or positive post-5^th^ IFN-γ, IL2 and IFN-γ+IL2 SPZ responses were included as shown in box. The ratios of positive post-5^th^: post-4^th^ IFN-γ, IL2 and IFN-γ+IL2 responses were determined in protected and non-protected subjects. When responses were unchanged the ratio was 1.0, a value chosen as an arbitrary cut off. IFN-γ (green) circles, IL2 (blue diamonds) and IFN-γ+IL2 (red squares); Cohort 1 (closed symbols) and Cohort 2 (open symbols). **Protected subjects:** **IFN-γ**: 11/12 (92%) were >1.0^1^; **IL2**: 10/14 (71%) were >1.0, **IFN-γ+IL2**: 7/7 (100%) were >1.0. **Non-protected subjects**: **IFN-γ**: 3/4 (75%) were <1.0, **IL2**: 4/5 (80%) were >1.0; **IFN-γ+IL2**: 3/3 (100%) were <1.0. ^1^One protected subject had a value of 1.0

**S1 Table. Demographic composition of Cohort 1 and Cohort 2**

|  | **Cohort 1** | **Cohort 2** | **Total** |
| --- | --- | --- | --- |
| **Number of Subjects** |  |  |  |
| **Gender** |  |  |  |
| Male | 20 (71%) | 20 (77%) | 40 (74%) |
| Female | 8 (29%) | 6 (23%) | 14 (26%) |
| **Total** | 28 | 26 | 54 |
| **Age (Years)** |  |  |  |
| 18-19 | 2 (7%) | 0 | 2 (4%) |
| 20-29 | 14 (50%) | 21 (81%) | 35 (64%) |
| 30-39 | 12 (43%) | 3 (12%) | 15 (28%) |
| 40-49 | 0 | 2 (7%) | 2 (4%) |
| 50 | 0 | 0 | 0 |
| **Average** | 28.0 | 27.5 | 27.8 |
| **Race** |  |  |  |
| Am. Indian/Alaska N. | 0 | 0 | 0 |
| Asian | 1 (4%) | 1 (4%) | 2 (4%) |
| Black or African Am. | 2 (7%) | 9 (35%) | 11 (20%) |
| Nat. Hawaiian/Other Pacific Is. | 0 | 0 | 0 |
| White | 20 (71%) | 16 (61%) | 36 (67%) |
| Other | 3 (11%) | 0 | 3 (5%) |
| Multiple^a^ | 2 (7%) | 0 | 2 (4%) |
| **Total** | 28 | 26 | 54 |
| **Ethnicity** |  |  |  |
| Hispanic/Latino | 4 (14%) | 2 (8%) | 6 (11%) |
| Not Hispanic/Latino | 21 (75%) | 21 (80%) | 42 (78%) |
| Not reported | 2 (7%) | 2 (8%) | 4 (7%) |
| Unknown | 1 (4%) | 1 (4%) | 2 (4%) |
| **Total** | 28 | 26 | 54 |
| **Military Member** |  |  |  |
| No | 21 (75%) | 17 (65%) | 38 (70%) |
| Yes | 7 (25%) | 9 (35%) | 16 (30%) |
| **Total** | 28 | 26 | 54 |

^a^ “Multiple” refers to subjects who identify with 2 or more race groups. See reference 14 for further details.

**S2 Table. Cohort 1: Summary of IFN-γ, IL2 and IFN-γ + IL2 responses recalled by whole sporozoites**

| **Cohort** |  | **No.** | **Cytokine** | **Immunization number** | | | | | |  |
| --- | --- | --- | --- | --- | --- | --- | --- | --- | --- | --- |
|  |  |  |  | **1*** | **1** | **2** | **3** | **4** | **5** | **Post-CHMI** |
| **1** | **All** | **11** | **IFN-γ** | 52 (8-502) | 75 (8-448) | 51 (3-229) | 44 (4-196) | 29 (1-92) | 41 (5-278) | 53 (8-172)^2^ |
|  |  |  | **No. +ve^1^** | 8 (73%) | 9 (82%) | 7 (64%) | 8 (73%) | 7 (64%) | 7 (64%) | 7^1^ (58%) |
|  |  |  | **IL2** | 37 (3-291) | 160 (29-654) | 95 (5-331) | 97 (32-417) | 74 (15-208) | 90 (16-445) | 84 (22-236)^2^ |
|  |  |  | **No. +ve** | 8 (73%) | 10 (91%) | 9 (82%) | 10 (91%) | 8 (73%) | 9 (82%) | 9^1^ (82%) |
|  |  |  | **IFN-γ +IL2** | 9 (1-128) | 31 (3-195) | 15 (1-83) | 15 (1-64) | 11 (1-33) | 17 (4-103) | 18 (3-59)^2^ |
|  |  |  | **No. +ve** | 2 (18%) | 7 (64%) | 7 (64%) | 5 (45%) | 4 (36%) | 5 (45%) | 3^1^ (27%) |
|  | **P** | **6** | **IFN-γ** | 49 (33-66) | 74 (8-223) | 51 (3-229) | 48 (5-196) | 40 (9-92) | 64 (13-278) | 54 (14-172) |
|  |  |  | **No. +ve** | 5 (83%) | 5 (83%) | 4 (67%) | 4 (67%) | 4 (67%) | 4 (67%) | 4 (67%) |
|  |  |  | **IL2** | 51 (30-98) | 178 (29-513) | 107 (12 -331) | 112 (29-417) | 87 (18-208) | 131 (41-445) | 108 (33-236) |
|  |  |  | **No. +ve** | 6 (100%) | 5 (83%) | 5 (83%) | 6 (100%) | 4 (67%) | 6 (100%) | 6 (100%) |
|  |  |  | **IFN-γ +IL2** | 11 (6-21) | 32 (3-101) | 18 (1-70) | 16 (1-64) | 13 (3-30) | 28 (8-103) | 22 (4-59) |
|  |  |  | **No. +ve** | 0 (0%) | 4 (67%) | 4 (67%) | 3 (50%) | 1 (17%) | 4 (67%) | 3 (50%) |
|  | **NP** | **5** | **IFN-γ** | 55 (8-502) | 75 (14-448) | 51 (5-217) | 39 (4-121) | 20 (1-88) | 25 (5-77) | 51 (8-113)^2^ |
|  |  |  | **No. +ve** | 3 (60%) | 4 (80%) | 3 (60%) | 4 (80%) | 3 (60%) | 3 (60%) | 3^1^ (60%) |
|  |  |  | **IL2** | 26 (3-291) | 140 (33-654) | 82 (5-288) | 81 (32-188) | 61 (15-151) | 57 (16-117) | 58 (22-163)^1^ |
|  |  |  | **No. +ve** | 2 (40%) | 5 (100%) | 4 (80%) | 4 (80%) | 4 (80%) | 3 (60%) | 3^1^ (75%) |
|  |  |  | **IFN-γ +IL2** | 7 (1-128) | 29 (4-195) | 13 (1-83) | 13 (2-39) | 10 (1-33) | 10 (4-28) | 13 (3-38) |
|  |  |  | **No. +ve** | 2 (40%) | 3 (60%) | 3 (60%) | 2 (40%) | 3 (60%) | 1 (20%) | 0 (%) |

Responses were measured one week (1*) and four weeks (1) after the first immunization, four weeks after the second (2), third (3), and fourth (4) immunizations, 22-24 days after the fifth immunization (5) and 39-42 days after CHMI (Post-CHMI). Values are the geometric mean of responses of all subjects, protected (P) and non-protected (NP) in Cohort 1 and the range of responses (lowest to highest). The numbers of subjects with positive responses were defined as described in Methods. ^1^Indicates the number of subjects with positive responses, and per cent of subjects tested at that time point. ^2^Post-CHMI activity of one immunized subject was not measured.

**S3 Table. Cohort 2: Summary of IFN-γ, IL2 and IFN-γ + IL2 responses recalled by whole sporozoites**

| **Cohort** |  | **No.** | **Cytokine** | **Immunization number** | | | | |  |
| --- | --- | --- | --- | --- | --- | --- | --- | --- | --- |
|  |  |  |  | **1** | **2** | **3** | **4** | **5** | **Post-CHMI** |
| **2** | **All** | **10** | **IFN-γ** | 13 (1-43) | 14 (3-44) | 32 (4-81) | 19 (1-47) | 50 (28-143) | 65 (18-123)^2^ |
|  |  |  | **No. +ve^1^** | 5 (50%) | 2 (20%) | 6 (60%) | 5 (50%) | 8 (80%) | 8^1^ (89%) |
|  |  |  | **IL2** | 34 (1-148) | 26 (5-98) | 46 (1-141) | 38 (1-101) | 80 (19-239) | 99 (4-218)^2^ |
|  |  |  | **No. +ve** | 8 (80%) | 6 (60%) | 7 (70%) | 9 (90%) | 9 (90%) | 6^1^ (67%) |
|  |  |  | **IFN-γ +IL2** | 8 (1-16) | 5 (1-22) | 11 (1-31) | 8 (1-20) | 18 (5-60) | 17 (1-58)^2^ |
|  |  |  | **No. +ve** | 0 | 0 | 3 (30%) | 0 | 3 (30%) | 3^1^ (33%) |
|  | **P** | **9** | **IFN-γ** | 16 (1-43) | 13 (3-44) | 32 (4-81) | 18 (1-47) | 52 (28-111) | 68 (18-123)^2^ |
|  |  |  | **No. +ve** | 5 (56%) | 1 (11%) | 6 (67%) | 4 (44%) | 7 (78%) | 8^1^ (88%) |
|  |  |  | **IL2** | 29 (1-148) | 22 (5-98) | 37 (1-141) | 32 (1-101) | 61 (19-239) | 94 (17-218) |
|  |  |  | **No. +ve** | 7 (78%) | 5 (56%) | 7 (78%) | 8 (89%) | 8 (89%) | 6^1^ (75%) |
|  |  |  | **IFN-γ +IL2** | 6 (1-16) | 5 (1-22) | 9 (1-31) | 7 (1-20) | 14 (5-60) | 24 (4-58)^2^ |
|  |  |  | **No. +ve** | 0 | 0 | 3 (33%) | 0 | 3 (33%) | 3^1^ (38%) |
|  | **NP** | **1** | **IFN-γ** | 47 | 32 | 29 | 52 | 38 | 47 |
|  |  |  | **No. +ve** | 1 (100%) | 1 (100%) | 0 | 1 (100%) | 1 (100%) | 1 (100%) |
|  |  |  | **IL2** | 60 | 43 | 34 | 53 | 43 | 4 |
|  |  |  | **No. +ve** | 1 (100%) | 1 (100%) | 0 | 1 (100%) | 1 (100%) | 0 |
|  |  |  | **IFN-γ +IL2** | 15 | 5 | 7 | 14 | 19 | 0 |
|  |  |  | **No. +ve** | 0 | 0 | 0 | 0 | 0 | 0 |

Responses (spot forming cells/million PBMC, sfc/m) were measured four weeks (1) after the first immunization, four weeks after the second (2), third (3), and fourth (4) immunizations, 22-24 days after the fifth immunization (5) and 39-42 days after CHMI (Post-CHMI). Values are the geometric mean of responses of all subjects, protected (P) and non-protected (NP), in Cohort 2 and the range of responses (lowest to highest). The numbers of subjects with positive responses were defined as in Methods. ^1^Indicates the number of subjects with positive responses, and per cent of subjects tested at that time point. ^2^Post-CHMI activity of one immunized subject was not measured.

**S4 Table. Cohorts 1 and 2: fold changes of IFN-γ, IL2 and IFN-γ + IL2 responses post-4^th^ and post-5^th^ immunizations**

| **COHORT 1** | **Protected** | | | **Fold** |  | **COHORT 1** | **Non-protected** | | | **Fold** |
| --- | --- | --- | --- | --- | --- | --- | --- | --- | --- | --- |
| **Cytokine** | **Subject** | **sfc/m^1^** | **sfc/m^2^** | **2:1** |  | **Cytokine** | **Subject** | **sfc/m^1^** | **sfc/m^2^** | **2:1** |
| **IFN-γ** | **2** | **87** | **124** | 1.43 |  | **IFN-γ** | **5** | **64** | **77** | 1.20 |
|  | **39** | **70** | **78** | 1.11 |  |  | **21** | **53** | **47** | 0.89 |
|  | **45** | 16 | 13 |  |  |  | **33** | 11 | 5 |  |
|  | **57** | **92** | **278** | 3.02 |  |  | **61** | **88** | **30** | 0.34 |
|  | **60** | **49** | **90** | 1.84 |  |  | **71** | 1 | 18 |  |
|  | **78** | 9 | 23 |  |  |  |  |  |  |  |
| **IL2** | **2** | **173** | **128** | 0.74 |  | **IL2** | **5** | **115** | **96** | 0.63 |
|  | **39** | **208** | **238** | 1.14 |  |  | **21** | **151** | **117** | 0.77 |
|  | **45** | 49 | **41** |  |  |  | **33** | **40** | 40 |  |
|  | **57** | **137** | **445** | 3.25 |  |  | **61** | **83** | 16 |  |
|  | **60** | **100** | **175** | 1.75 |  |  | **71** | 15 | **87** |  |
|  | **78** | 18 | 53 |  |  |  |  |  |  |  |
| **IFNγ+IL2** | **2** | **30** | **35** | 1.17 |  | **IFN-γ+IL2** | **5** | **33** | **28** | 0.85 |
|  | **39** | 17 | **37** |  |  |  | **21** | **30** | **21** | 0.70 |
|  | **45** | 6 | 8 |  |  |  | **33** | 8 | 4 |  |
|  | **57** | 20 | **103** |  |  |  | **61** | **28** | **7** | 0.25 |
|  | **60** | 19 | **42** |  |  |  | **71** | 1 | 8 |  |
|  | **78** | 3 | 11 |  |  |  |  |  |  |  |
| **Cohort 2** | **Subject** | **sfc/m^1^** | **sfc/m^2^** | **2:1** |  | **Cytokine** | **Subject** | **sfc/m^1^** | **sfc/m^2^** | **2:1** |
|  | **102** | **47** | **76** | 1.62 |  |  | **110** | **52** | **38** | 0.73 |
| **IFN** | **103** | **38** | 28 |  |  | **IFN** |  |  |  |  |
|  | **114** | 11 | **29** |  |  |  |  |  |  |  |
|  | **120** | 1 | 25 |  |  |  |  |  |  |  |
|  | **127** | 23 | **54** |  |  |  |  |  |  |  |
|  | **130** | 20 | **143** |  |  |  |  |  |  |  |
|  | **131** | **43** | **43** | 1 |  |  |  |  |  |  |
|  | **133** | **40** | **111** | 2.78 |  |  |  |  |  |  |
|  | **134** | 14 | **51** |  |  |  |  |  |  |  |
| **IL2** | **102** | **93** | **118** | 1.27 |  | **IL2** | **110** | 53 | 43 | 0.81 |
|  | **103** | **76** | **43** | 0.57 |  |  |  |  |  |  |
|  | **114** | **50** | **86** | 1.72 |  |  |  |  |  |  |
|  | **120** | 1 | 19 |  |  |  |  |  |  |  |
|  | **127** | **58** | **103** | 1.78 |  |  |  |  |  |  |
|  | **130** | **53** | **233** | 4.4 |  |  |  |  |  |  |
|  | **131** | **44** | **36** | 0.82 |  |  |  |  |  |  |
|  | **133** | **101** | **239** | 2.37 |  |  |  |  |  |  |
|  | **134** | **33** | **83** | 2.52 |  |  |  |  |  |  |
| **IFN+IL2** | **102** | 20 | **33** | 1.65 |  | **IFN+IL2** | **110** | 14 | 19 |  |
|  | **103** | 13 | 8 |  |  |  |  |  |  |  |
|  | **114** | 6 | 14 |  |  |  |  |  |  |  |
|  | **120** | 1 | 5 |  |  |  |  |  |  |  |
|  | **127** | 10 | 24 |  |  |  |  |  |  |  |
|  | **130** | 6 | **46** |  |  |  |  |  |  |  |
|  | **131** | 14 | 8 |  |  |  |  |  |  |  |
|  | **133** | 18 | **60** |  |  |  |  |  |  |  |
|  | **134** | 4 | 15 |  |  |  |  |  |  |  |

Responses (spot forming cells/million PBMC, sfc/m) were measured four weeks after the fourth immunizations and 22-23 days after the fifth immunizations. Positive responses are shown in bold red. The fold increase of responses after the fourth and fifth immunizations are shown as Fold, and only include subjects with positive responses post-4^th^ and post-5^th^ immunizations.
